# Sociodemographics, Clinical Factors, and Biological Factors Associated with Loiasis in Endemic Onchocerciasis Areas in Southern Gabon

**DOI:** 10.4269/ajtmh.22-0558

**Published:** 2023-06-20

**Authors:** Reinne Moutongo Mouandza, Jean Romain Mourou, Bridy Moutombi Ditombi, Hadry Roger Sibi Matotou, Bernadette Ekomi, Marielle Karine Bouyou-Akotet, Denise Patricia Mawili-Mboumba

**Affiliations:** Department of Parasitology-Mycology and Tropical Medicine, Faculty of Medicine, Université des Sciences de la Santé, Libreville, Gabon

## Abstract

To implement the appropriate strategies for scale-up interventions to eliminate onchocerciasis without severe adverse events, clinical and biological factors associated with loiasis were analyzed in onchocerciasis-endemic areas. Blood was collected from volunteers after examination by a physician. Detection of microfilariae and measurement of Ov16 IgG4 were performed using direct microscopic examination of blood and onchocerciasis rapid test detection, respectively. Areas with sporadic, hypoendemic, and hyperendemic onchocerciasis endemicity were found. Participants with microfilaremia were considered microfilaremic, and those without microfilaremia were seen as amicrofilaremic. Of the 471 study participants, 40.5% (*n* = 191) had microfilariae. Among them, *Mansonella *spp. was the most common (78.2%, *n* = 147), followed by *Loa loa* (41.4%, *n* = 79). The association between the two species represented 18.3% (*n* = 35). The specific immunoglobulins of *Onchocerca volvulus* were detected in 24.2% of participants (*n* = 87/359). Overall prevalence of *L. loa* was 16.8%. Hypermicrofilaremia was found in 3% (*N* = 14), and one participant had more than 30,000 microfilaremiae per milliliter. The frequency of *L. loa* did not vary according to the level of onchocerciasis transmission. Pruritus was the most common clinical sign (60.5%, *n* = 285) reported, mainly in microfilaremic participants (72.2%, *n* = 138/191). The prevalence of *L. loa* microfilaria in the study population was below the threshold at risk for the occurrence of serious side effects due to ivermectin. Clinical manifestations frequently observed could be exacerbated by microfilaremia in areas where onchocerciasis transmission is high.

## BACKGROUND

Loiasis is a chronic disease caused by *Loa loa* filarial species prevalent in central and west Africa.^1^ Adult worms can live in subcutaneous tissues for up to 17 years and produce microfilariae in peripheral blood. This disease is often characterized by mild clinical symptoms in people living in endemic areas. The most common clinical manifestations are itching, angioedema on the arms known as a Calabar swelling, and ocular migration of the adult worm under the conjunctiva.^2^^–^^4^ Onchocerciasis may also be detected in central African individuals living in loiasis-endemic areas such as Gabon. According to the WHO, ∼198.2 million people are at risk of onchocerciasis, and 99% of them reside in Africa.^5^ Onchocerciasis is of public health importance and is associated with blindness, vision problems, skin disease, and itching. Onchocerciasis is controlled by repeated mass drug administration (MDA) in Africa, based on annual doses of ivermectin.^6^^,^^7^ However, treatment programs of onchocerciasis have been interrupted in areas where the diseases are co-endemic. Indeed, after onchocerciasis treatment, serious adverse events (SAEs) such as encephalopathy may occur in patients with high *L. loa* microfilaremia.^8^^,^^9^ The severity of the symptoms may be even greater when the density of *L. loa* microfilariae exceeds 8,000 microfilariae per milliliter.^8^ To identify an area at risk of SAEs related to *L. loa* infection, the Rapid Assessment Procedure for *L. loa* (RAPLOA) was developed over the past decade.^10^^,^^11^ This assessment is based on the prevalence of the history of adult worms’ migration under the conjunctiva of the eye.^11^^,^^12^ In endemic areas, some individuals present few or nonspecific symptoms and are at risk of developing SAEs in the absence of loiasis diagnosis before MDA.^13^^,^^14^ In onchocerciasis hypoendemic areas such as Gabon, the prevalence of *L. loa* microfilariae varies between 4.7% and 39.5%. In addition to *L. loa*, other filarial species such as *Mansonella *spp. are endemic, and some clinical symptoms could be associated with this species.^15^ In 2015, a potential new species of *Mansonella* called *Mansonella *sp. DEUX was reported in febrile children.^16^ Recently, this species has been the most frequently identified filarial nematode (74%) by molecular technique.^17^ To allow the establishment of criteria necessary to distinguish persons at risk of MDA ivermectin SAEs, investigation and identification of the factors associated with *L. loa* microfilaremiae are required to implement the appropriate strategies to scale up interventions for the elimination of *Onchocerca volvulus* without the occurrence of SAEs. The aim of our study was to analyze demographics and the clinical and biological profile of the *L. loa* microfilaremic population living in areas of onchocerciasis transmission.

## MATERIALS AND METHODS

### Study area and population.

This study was conducted in five onchocerciasis communities across Gabon. The study sites are located in southern Gabon, specifically in the department of Louétsi-Wano (Ngounié province), ∼650 km from Libreville, the capital city of Gabon. Its population is estimated at 9,750 inhabitants. The main city of this department is Lébamba, and the five communities selected around that city are covered by rainforest and located 4 to 40 km far from Lébamba: Memba (4 km), Nzoundou-Issinga (10–14 km), Mayanga (20 km), Mbelnaletembe (28 km), and Matamatsengue-Nzingui (38–40 km). In 2014, onchocerciasis prevalence was 17.3% in Memba, 59.0% in Nzoundou-Issinga, and 33.9% in Mbelnaletembe according to a National Parasitic Disease Control Program survey.^18^^,^^19^ Since then, no mass treatment has been undertaken in these communities. The individual treatment is done after medical consultation by a specialist. The National Parasitic Disease Control Program recommends ivermectin for the treatment of onchocerciasis and loiasis in cases of low microfilaremia (< 8,000/mL). The high density of *L. loa* is treated with albendazole to reduce microfilarial loads before ivermectin administration. In Lébamba, people receive their medical care at the city’s medical center. In each selected community, the population receives care in their respective smaller healthcare centers, apart from Mayanga, where the healthcare center was closed at the time of the survey. The population’s main activities in these communities are agriculture, hunting, and fishing. All residents over 5 years old were invited to take part in the study protocol. After explaining the purpose of the study, the people who gave their consent to participate were interviewed using a structured questionnaire. Sociodemographics, living conditions, and medical history data were recorded. Volunteers received a complete physical examination by the study team physician. Identified clinical symptoms were recorded on the report form.

### Sample size calculation.

The required sample size was determined as follows: *n* = Z^2^ × P (1 − P)/d^2^, where *n* = sample size, P = expected prevalence, d = precision (at 5% marginal error), and Z = standard score at 95% CI.^20^ The prevalence of loiasis, estimated on the basis of previously reported data in Ngounié province, was 30.5% before the present study.^21^ The minimum sample size determined for the study was *N* = 326.

### Biological sample collection.

Blood (4 mL) was collected in EDTA tubes between 10:00 am and 3:00 pm because of the *L. loa* daytime periodicity. Each tube was preidentified with an ID number matching that of the participant. Filariasis diagnosis was determined as previously reported.^22^ Participants with more than one filaria species identified were considered as coinfected.

### Microfilariae detection.

The detection of microfilariae was performed with a direct microscopic examination of 10 µL of fresh uncoagulated blood. All the microfilariae present in the slide were identified and counted. Parasitaemia was expressed as the number of microfilariae per milliliter (mf/mL) of blood. The limit of microfilaria detection with this method is 100 mf/mL. All participants with a *L. loa* microfilaria count > 8,000 were considered as hypermicrofilaremic. Those without microfilaremia were labeled amicrofilaremic. Those who had *L. loa* microfilaremia density < 8,000 were considered as low microfilaremic.

### Onchocerciasis diagnosis and classification.

Detection of onchocerciasis circulating antibodies was performed using the Ov16 rapid diagnostic test SD Bioline (Abbott Standard Diagnostics, Inc., Yongin, South Korea) according to the manufacturer’s instructions. The results were available 20 minutes later. In view of concordance between the prevalence of positive Ov16 rapid diagnostic test (RDT) and that found by skin snip during the survey previously conducted by the National Parasitic Disease Control Program, the onchocerciasis endemicity was classified as sporadic for villages where the frequency of positive RDT was below 10%, hypoendemic when the positivity rate was between 10% and 35%, and hyperendemic when it was higher than 60%.^18^

### Quality control of readings.

Quality control of the slides and Ov16 RDT reading was performed by two independent technicians, unaware of the reading of each other. In cases of discordance, a third technician read the slides and Ov16 RDT.

### Statistical analysis.

The data were recorded in a spreadsheet and analyzed using the Stat view 5.0 software (SAS Institute, Cary, NC). Medians are presented with interquartile range in brackets. Statistical analysis for group comparison was performed using χ^2^ and Fisher exact tests. Quantitative data were compared using nonparametric tests and analysis of variance. Statistical significance was set at *P* < 0.05 for all analysis.

## RESULTS

During the study period, 471 participants from five communities were included. Ov16 RDT was used to estimate the level of onchocerciasis transmission in each community. Of the 359 RDT performed, 24.2% (*n* = 87) were positive. Three onchocerciasis levels of transmission were defined: sporadic for Memba; hypoendemic for Mbelnaletembe, Nzingui/Matamatsengue, Mayanga; and hyperendemic for Issinga/Nzoundou (Table 1). More than 50% of the participants (59.0%, *n* = 278) came from hypoendemic onchocerciasis areas, followed by sporadic (27.6%, *n* = 130) and hyperendemic areas (13.4%, *n* = 63). The sex ratio was 0.93. Almost two-thirds of the participants did not complete primary school, and one quarter did not attend school—63.5% (*N* = 299) and 24.2% (*N* = 114), respectively. The population was mainly unemployed (95%).

**Table 1:** Frequency of onchocerciasis Ov16 Ig4 seropositivity and transmission classification

Communities	RDT performed	RDT positive	% [95% CI]	Endemicity type
Memba	82	4	4.8 [1.9–11.9]	Sporadic
Mbelnaletembe	56	8	14.2 [7.4–25.7]	Hypoendemic
Nzingui/Matamatsengue	127	25	20.0 [13.9–27.9]	Hypoendemic
Issinga/Nzoundou	62	43	69.4 [57.1–79.4]	Hyperendemic
Mayanga	32	7	21.9 [11.0–38.8]	Hypoendemic

RDT = rapid diagnosis test.

### Study participants’ characteristic distribution according to onchocerciasis transmission level.

In the different onchocerciasis endemic areas, the population did not differ according to sex (*P* = 0.408). However, females represented more than 50% of the study population in sporadic and hypoendemic areas, whereas males accounted for 56.6% in hyperendemic areas (Table 2). The patients were younger in hypoendemic onchocerciasis transmission areas, where 49.3% were aged less than 20 years. Those older than 45 years were more frequent in hyperendemic (42.8%) and sporadic (41.9%) areas (*P* = 0.0035) (Table 2). More than half of the participants had a primary school level education: 57.7%, 66.9%, and 58.7% in sporadic, hypoendemic, and hyperendemic areas, respectively (*P* < 0.0019) (Table 2).

**Table 2:** Study participants’ sociodemographic characteristic according to onchocerciasis transmission level

Characteristic	Onchocerciasis transmission level			
	Sporadic, *n* (%)	Hypoendemic, *n* (%)	Hyperendemic, *n* (%)	% [95% CI]
Gender (*N* = 471)				
Male	59 (45.4)	133 (47.8)	35 (55.6)	48.2 [43.7–52.5]
Female	71 (54.6)	145 (52.2)	28 (44.4)	51.8 [47.5–56.3]
Age in years* (*N* = 464)				
< 20	55 (42.6)	134 (49.3)	18 (28.6)	43.3 [38.5–48.3]
21–45	20 (15.5)	62 (22.8)	18 (28.6)	20.9 [17.4–24.1]
> 45	54 (41.9)	76 (27.9)	27 (42.8)	32.8 [24.8–37.0]
School attendance (*N* = 471)				
None or preschool	33 (25.4)	71 (25.5)	11 (17.5)	24.2 [20.4–28.5]
Primary school	75 (57.7)	186 (66.9)	37 (58.7)	63.5 [59.0–68.0]
Secondary school	22 (16.9)	21 (7.6)	15 (23.8)	12.3 [9.6–15.3]

* Age was unknown for seven participants.

### *Loa loa* infection distribution according to sex, age, school attendance, and onchocerciasis transmission level.

Microfilaria were identified in 191 participants (40.5%). Among them 41.4% (*n* = 79/191) had *L. loa* microfilaria, and the prevalence in overall participants was 16.8%. The median *L. loa* density was 800 (interquartile range 200–5200) mf/mL. One participant had more than 30,000 mf/mL (0.2%). However, *Mansonella *spp. was the most common species (77.0%; *n *= 147/191). This species was twice more frequent in *L. loa* microfilaremic participants (44.3%; *n* = 35/79) than in individuals without *L. loa* microfilaremia (28.6%; *n* = 112/392). Less than one-quarter of the infected participants (18.3%; *n* = 35/191) were coinfected by *L. loa* and *Mansonella *spp. species.

The distribution of *L. loa*–infected patients was not statistically different according to the level of onchocerciasis transmission: 14.3% in hyperendemic, 16.9% in sporadic, and 17.3% in hypoendemic onchocerciasis areas (*P* = 0.848) (Table 2). The prevalence of low microfilaremia was comparable in the three onchocerciasis areas: 13.8% (*n* = 18/130) in sporadic, 14.0% (*n* = 39/278) in hypoendemic, and 12.7% (*n* = 8/63) in hyperendemic areas. Hypermicrofilaremia frequency was low in all areas (3.1%, *n* = 4/130 and 3.2%, *n* = 9/278 in sporadic and hypoendemic areas, respectively; *P* = 0.782). In the onchocerciasis hyperendemic area, one (1.6%, *n* = 1/63) participant had *L. loa* hypermicrofilaremia.

With reference to the sex, males were not more frequently infected by *L. loa* (15.2% versus 18.5% in women) (Table 3).

**Table 3:** *Loa loa* infection distribution according to onchocerciasis transmission level, age, gender, and school attendance

Characteristics	No *L. loa* microfilaremia, *n *(%)	*L. loa* microfilaremia, *n* (%)	*P*
Onchocerciasis transmission			
Sporadic	108 (83.1)	22 (16.9)	0.8480
Hypoendemic	230 (82.7)	48 (17.3)	
Hyperendemic	54 (85.7)	9 (14.3)	
Gender			
Male	185 (84.8)	42 (15.2)	0.3362
Female	207 (81.5)	37 (18.5)	
Age range (years)			
< 20	196 (94.7)	11 (5.3)	< 0.00001
21–45	83 (83.0)	17 (17.0)	
> 45	107 (68.2)	50 (31.8)	
School attendance			
None or preschool	82 (71.3)	33 (28.7)	< 0.00006
Primary school	264 (88.6)	34 (11.4)	
Secondary school	45 (77.6)	13 (22.4)	

**Figure 1.**
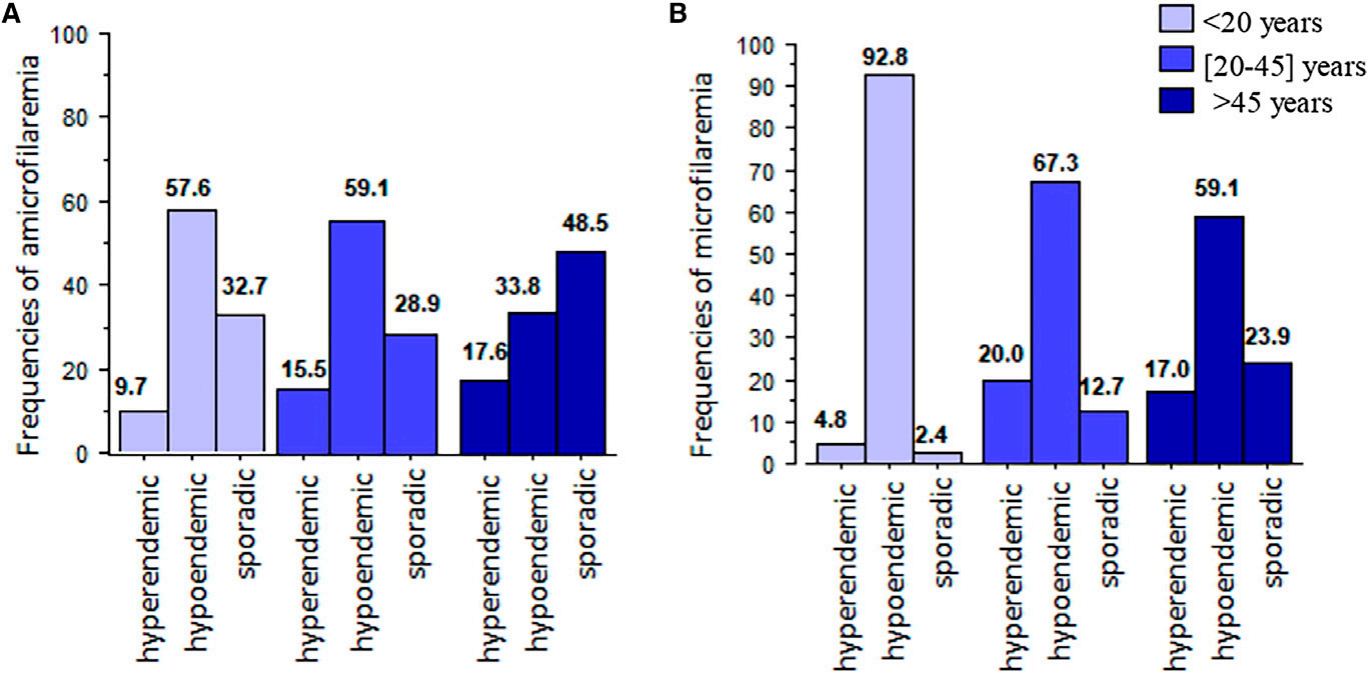
Relation between microfilaremia and range age according to level of onchocerciasis transmission. (**A**) Frequencies of amicrofilaremic participants according to range age in areas with different level of onchocerciasis transmission. (**B**) Frequencies of microfilaremic participants according to range age in areas with different level of onchocerciasis transmission.

The frequency of infected participants increased significantly with age, the oldest (older than 45 years) being the more frequently infected by *L. loa* microfilariae (*P* < 0.001) (Table 3). The elation between microfilaremia and range age according to level of onchocerciasis transmission was described in Figures 1A and B. Hypermicrofilaremia was significantly more frequent in participants older than 45 years (5.7%; *n* = 9/157) compared with those younger than 20 years (0.48%, *n* = 1/207; *P* < 0.001). Most of microfilaremic participants younger than 20 years (92.8%, *n* = 39/42) lived in hypoendemic onchocerciasis areas (Figure 1B). In those areas, the frequencies of microfilaremic participants were similar among individuals older than 45 years and those aged 20 to 45 years: 59.5% (*n* = 53/89) and 67.3% (*n* = 37/55) respectively (*P* = 0.36). In sporadic onchocerciasis areas, participants older than 45 years were less frequently infected compared with those aged from 20 to 45 years living in the same area (23.6%, *n* = 21/89 and 12.7%, *n* = 7/55), although this was not statistically significant (*P* = 0.11) (Figure 1B).

### Prevalence of clinical symptoms related to *L. Loa* and *Mansonella *spp. microfilaremia according to onchocerciasis transmission level.

**Figure 2.**
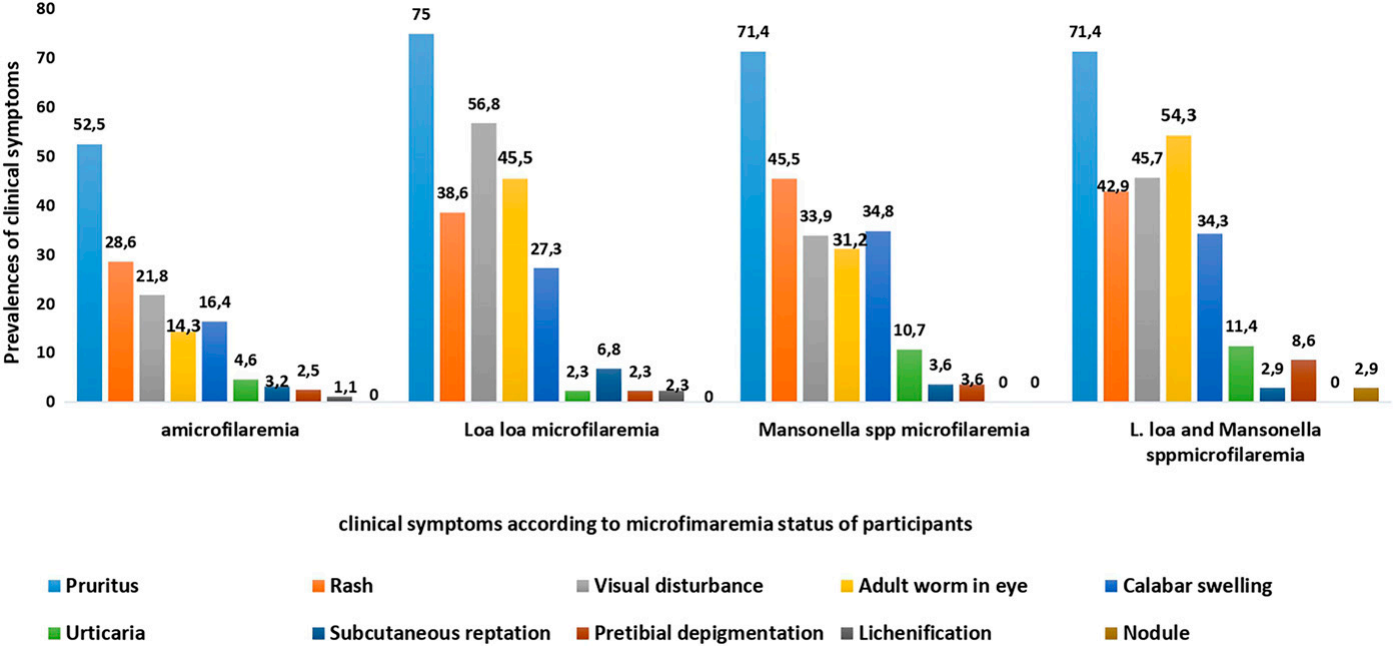
Relation between clinical symptoms and *Loa loa* and/or *Mansonella *spp. microfilaremia.

**Figure 3.**
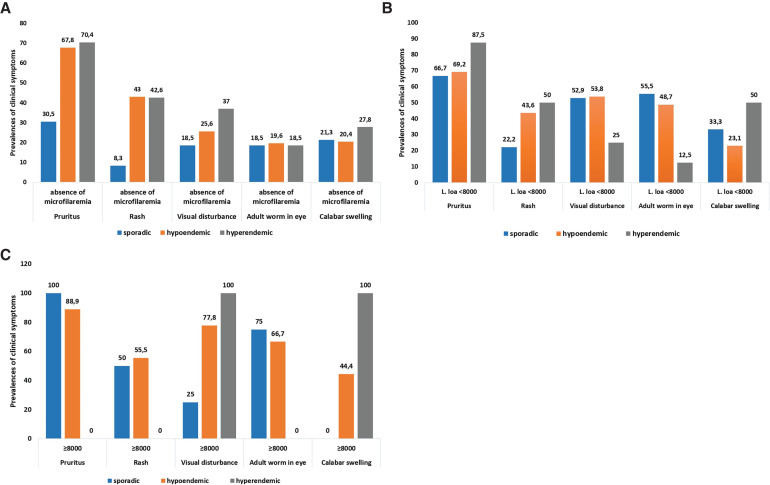
Relation between clinical signs and *Loa loa* microfilaremia densities according to level of onchocerciasis endemicity. (**A**) Distribution of clinical signs of participants without microfilaremia according to onchocerciasis endemicity areas. (**B**) Distribution of clinical signs of patients with low *L. loa* microfilaremia according to onchocerciasis endemicity areas. (**C**) Distribution of clinical signs of patients with high *L. loa* microfilaremia according to onchocerciasis endemicity areas.

Pruritus was the most common symptom (60.5%, *n* = 285) in the study participants followed by rash (34.6%, *n* = 163) and visual disturbance (27.9%, *n* = 140). Adult worm in the eye and Calabar swelling were found in 24.2% (*n* = 114) and 23.1% (*n* = 109) of the cases, respectively. One patient had nodules. Clinical symptoms were more frequently seen in microfilaremic participants than in those without microfilaremia (*P* < 0.001). Pruritus was highly prevalent in *L. loa* microfilaremic participants (75.0%, *n* = 33/44) and in *Mansonella *spp. microfilaremic participants (71.4%, *n* = 80/112), as well as in those infected by both species (71.4%, *n* = 25/35) compared with amicrofilaremic participants (Figure 2) (*P* < 0.001). Visual disturbance and adult worm in the eye were significantly more frequent in patients with *L. loa* microfilaremia (56.8%, *n* = 25/44 and 45.5%, *n* = 20/44, respectively) and those coinfected with the two species (45.7%, *n* = 16/35 and 54.3%, *n* = 19/35, respectively) compared with those having *Mansonella *spp. microfilaremia (33.9%, *n* = 38/112 and 31.2%, *n* = 35/112, respectively; *P* = 0.02) (Figure 2). Calabar swelling, rash and urticaria were more common in individuals infected with *Mansonella *spp. microfilaremia (Figure 2). The frequency of clinical symptoms tended to increase with *L. loa* microfilaremia density (Figure 3). Thus, the highest prevalence of pruritus was observed in hypermicrofilaremic participants living in sporadic (100%, *n* = 4) and hypoendemic onchocerciasis areas (88.9%, *n* = 8; *P* = 0.034) (Figure 3B). All hypermicrofilaremic participants living in hyperendemic onchocerciasis areas had Calabar swelling (*P* < 0.01) (Figure 3C). However, in participants with low microfilaremia, the frequency of adult worm in the eye decreased with the level of onchocerciasis endemicity without statistical difference (*P* > 0.05).

Considering the onchocerciasis clinical symptoms and microfilaremia, pretibial depigmentation was more frequent in patients infected with *L. loa* and *Mansonella *spp. species. Likewise, nodules were found in patients with coinfection only. Lichenification was identified in less than 3% (1.1%, *n* = 3/280) of patients in absence of microfilaremia or with *L. loa* microfilaremia (2.3%, *n* = 1/44) (Figure 3A).

Pruritus, rash, and visual disturbance were more frequently detected in individuals living in hypo and hyperendemic onchocerciasis areas (*P* < 0.01 and *P* < 0.02) (Table 4). The presence of adult worm in the eye was more frequently reported in hypoendemic and hyperendemic onchocerciasis areas, although it was not statistically different according to the area (*P* = 0.461). When considering *L. loa* microfilaremia density among patients with more than 8,000 mf/mL, rash and adult worm in the eye were more frequently recorded in those living in sporadic and hypoendemic areas. Inversely, visual disturbance and Calabar swelling were found in all *L. loa*–infected patients living in hyperendemic areas, whatever the level of microfilaremia density (Figure 3).

**Table 4:** Relation between *Loa Loa* clinical symptoms and microfilaremia level according to onchocerciasis transmission level

Characteristic	Onchocerciasis transmission level			
	Sporadic, *n* (%)	Hypoendemic, *n* (%)	Hyperendemic, *n* (%)	*P*
Clinical symptoms				
Pruritus, < 0.01	49 (37.7)	191 (68.7)	45 (71.4)	–
Rash, < 0.01	15 (11.5)	121 (43.5)	27 (42.9)	–
Visual disturbance, 0.025	30 (23.3)	87 (31.3)	23 (36.5)	–
Adult worm in eye, 0.461	11 (17.5)	70 (25.2)	33 (25.4)	–
Calabar swelling, 0.125	29 (22.3)	60 (21.6)	20 (31.7)	–
Urticaria	2 (1.5)	25 (9.0)	3 (4.8)	0.021
Subcutaneous reptation	9 (6.9)	4 (1.4)	4 (6.3)	0.070
Pretibial depigmentation	0 (0.0)	9 (3.2)	6 (9.5)	0.020
*L. loa* densitity median, mf/mL				
Low microfilaremia	700 [200–2,400]	500 [225–2,375]	500 [200–1500]	0.5369
High microfilaremia	9,500 [8,550–14,400]	15,000 [8,900–21,850]	18,700*	0.3034

* One participant with high microfilaremia density was found in hyperendemic onchocerciasis areas.

## DISCUSSION

Three areas were identified in the study, according to their levels of onchocerciasis endemicity. Indeed, a wide heterogeneity of filariasis up to department levels in Gabon was reported.^18^ In these regions, almost half of the participants who lived in onchocerciasis-hypoendemic areas were children aged between 5 and 20 years. This result underlines that a nonnegligible proportion of children could be infected with *O. volvulus*, requiring the implementation of a disease control strategy that targets this part of the population. Onchocerciasis mainly affects adults, but there is increasing evidence that *O. volvulus* infection in children can lead to severe disorders such as onchocerciasis-associated epilepsy.^23^^,^^24^ Also, children, notably those under 5, may represent an important reservoir.^25^

Onchocerciasis, mansonellosis, and loiasis are co-endemic in our study areas, which constitutes a bottleneck for onchocerciasis morbidity control. In effect, our investigation reports that more than one-third of the participants were *L. loa* and/or *Mansonella *spp. microfilaremic (40.5%). More than three-quarters (77.0%) of the participants were infected with microfilariae had *Mansonella *spp. This result is in agreement with the finding of Sandri et al., who described the high prevalence of mansonellosis in this country.^17^

The overall prevalence of *L. loa* microfilaremia among the study participants (16.8%) is comparable to that previously reported (22.4% and 16.7%).^26^^,^^27^ In contrast, the prevalence of *Mansonella *spp. as single (23.8%, *n* = 112/471) and co-infection with *L. loa* filarial species (7.4%, *n* = 35/471) was higher compared with previous reports from the country (10.2% and 3.2%; 5.6% and 3.4%, respectively).^15^^,^^26^^,^^28^

The frequency of *L. loa* microfilaremia (< 20%) did not vary according to the level of onchocerciasis transmission in the communities, suggesting that the variation of loiasis is more related to the bioecological settings.^29^ Otherwise, a low prevalence of hypermicrofilaremia was observed whatever the level of onchocerciasis transmission; the same was also reported in a study carried out in other central African areas.^30^ The highest density of *L. loa* microfilariae was found in individuals living in hypoendemic onchocerciasis transmission areas. In those areas, only one case was reported. Recently, it was established that there is an association between *L. loa* microfilariae densities > 30,000 per milliliter and increased risk of mortality in people older than 25 years.^31^ Knowing that low prevalence of hypermicrofilaremia was found and only 0.2% of the patients had more than 30,000 microfilariae per milliliter in the present study, a systematic screening followed by MDA could be envisaged to reduce filariasis morbidity and prevent the risk of occurrence of encephalopathy. Equally relevant, albendazole pretreatment could be undertaken to reduce microfilarial density in the subject with high *L. loa* microfilaremia.^32^ For the purpose of this study, we used albendazole 400 mg per day, for 1 to 2 weeks, according to the loads of microfilaremia to treat participants who had > 8,000 per milliliter of *L. loa* rmicrofilaremia.

The prevalence of microfilaremic patients increased with age and was sex-specific, being more frequent in men.^33^^–^^35^ In contrast, men were not likely to have a higher prevalence of *L. loa* microfilaremia than women in the present study. Women living in this study area were exposed as frequently as men to the biting of vectors, mainly due to farming activities.

Concerning age, it is worth noting that the daily occupation of the study participants plays a role in exposure. As an example, daily activities such as working with food crops are mainly performed by the oldest, whereas the youngest attend school or are unexposed during other daily activities. Among the participants attending school, those in primary school were less infected. This group is essentially composed of children who are not involved in subsistence activities. Furthermore, the oldest participants had a higher density of *L. loa* microfilaremia than those aged less than 20 years. This result underlines a relationship between consecutive exposure to new infective bite by the vectors and intensity as previously described.^36^

Among the clinical manifestations, pruritus was the most common symptom, found in almost two-thirds of the study population. The finding that pruritus is more frequent in microfilaremic individuals than those without microfilaremia may be explained by onchocerciasis coinfection. Despite it being nonspecific to loiasis, the prevalence of this clinical symptom was reported in three-quarters of *L. loa* microfilaremic participants (75.0%) in the present study. Otherwise, visual disturbance (56.8%) and adult worm in the eye (45.5%) were the second and the third most common symptoms observed in *L. loa* microfilaremic participants in the present study. In a recent study showing the high burden of loiasis with substantial morbidity, adult worm in the eye was reported in 42.2% of eligible participants, with vision impairment affecting more than three-quarters of patients.^37^^,^^38^ Pruritus and Calabar swelling symptoms were more prevalent in *Mansonella *spp. microfilaremic participants (71% and 34.8%, respectively) in this study, compared with those previously shown by Bouyou Akotet et al. in the same country (28% and 8%, respectively).^15^ This discrepancy could be explained by a presumably higher proportion of occult loiasis in the study population. In any case, considering the high frequency of this species and the clinical manifestations, studies must be undertaken to determine morbidity associated with *Mansonella *spp. The analysis of clinical symptom frequencies according to the onchocerciasis endemicity showed that the frequency of pruritus, rash, and visual disturbance significantly increased with the level of onchocerciasis endemicity, mainly in participants with absence of microfilaremia. These results suggest an effect of onchocerciasis on the occurrence of clinical symptoms, as found previously.^39^^,^^40^ In addition, the prevalence of specific *O. volvulus* such as pretibial depigmentation and visual disturbance increased with the endemicity level. Thus, 3 times more cases of pretibial depigmentation were found in hyperendemic areas compared with hypoendemic areas (9.7% and 2.3%, respectively). In fact, a correlation between skin depigmentation and *O. volvulus* microfilarial density was established. This symptom could be a good marker of endemicity as suggested by Edungbola et al.^41^ Otherwise, the fact that the frequency of visual disturbance and adult worm in the eye decreased with the level of onchocerciasis transmission in low microfilaremic participants could reflect interaction between filarial species as suggested by Donohue et al. in their meta-analysis.^42^ Knowing that *L. loa* and *O. volvulus* live within host, these interactions could be related to the host immune cross-reaction.

More than 95% of the population in our study areas may benefit from MDA without risk of SAEs. Indeed, the low frequency of hypermicrofilaremiae and the high prevalence of patients without detectable *L. loa* microfilariae support this strategy after patient testing.

## CONCLUSION

Within areas with different levels of onchocerciasis endemicity, the frequencies of *L. loa* hypermicrofilaremia were low and did not vary. Pruritus, rash, visual disturbance, and pretibial depigmentation prevalence increased according to onchocerciasis endemicity. The prevalence of *L. loa* and the history of adult worm in the eye were below the threshold at risk for the occurrence of serious side effects after mass treatment with ivermectin as defined by RAPLOA. To reduce the burden of filariasis in these rural areas, MDA should be considered.
